# 2-deoxy-d-glucose Ameliorates Animal Models of Dermatitis

**DOI:** 10.3390/biomedicines8020020

**Published:** 2020-01-24

**Authors:** Soo Young Choi, Min-Jeong Heo, Chanmi Lee, Yeong Min Choi, In-sook An, Seunghee Bae, Sungkwan An, Jin Hyuk Jung

**Affiliations:** 1 Korea Institute of Dermatological Science, GeneCellPharm Corporation, 375 Munjeong 2(i)-dong, Songpa-gu, Seoul 05836, Korea; choisoo4563@skinresearch.or.kr (S.Y.C.); alselddlrh@skinresearch.or.kr (M.-J.H.); chanmi2026@skinresearch.or.kr (C.L.); sharmine@skinresearch.or.kr (Y.M.C.); anis@skinresearch.or.kr (I.-s.A.); 2 Research Institute for Molecular-Targeted Drugs, Department of Cosmetics Engineering, Konkuk University, Seoul 05029, Korea; sbae@konkuk.ac.kr

**Keywords:** 2-deoxy-d-glucose, dermatitis, TPA, oxazolone

## Abstract

Glucose metabolism is a key metabolic pathway that orchestrates cellular homeostasis by generating ATP, nucleotides, and amino acids. Abnormal glucose signaling has been found in many diseases including cancers and inflammatory diseases. According to recent report, glycolysis contributes to pathogenesis of psoriasis and ablation of Glut1 attenuates animal models of psoriasis. While we were screening a molecular target for atopic dermatitis, we found the levels of glucose transporters including Glut1 (*SLC2a1*) and Glut3 (*SLC2a3*) are highly expressed in skin biopsies of dermatitis patients from multiple datasets. We demonstrated that administration of 2-deoxy-d-glucose (2DG) ameliorates animal models of 12-o-tetradecanoylphorbol-13-acetate (TPA) and oxazolone induced dermatitis using morphological and histological analysis. These results suggest that inhibition of glucose metabolism ameliorates dermatitis in animal models.

## 1. Introduction

As glucose is the most frequently used carbon source for mammals, biological functions of glucose and glucose metabolism have been extensively studied for many decades [1,2,3]. Recent studies expanded the role of glucose into immunology, stem cell, and cancer biology [4,5,6,7]. For example, glucose and its metabolites regulate activation, development, and maintenance of immune cells [8,9]. Additionally, the metabolic interplay between glycolysis and mitochondria respiration serves an important role in stem cell differentiation [10]. Moreover, certain types of cancer exhibit glucose addiction, called the “Warburg effect”, which has been studied as a molecular target for anti-cancer treatment [11]. As 2-deoxy-d-glucose (2DG), a glucose analogue remains unmetabolized by hexokinase, it has been demonstrated to be powerful agent for blocking and probing increased sugar metabolism in cancer cells [12,13,14,15,16]. Dermatitis is a skin inflammatory disease, which includes atopic dermatitis and psoriasis [17]. Atopic dermatitis and psoriasis have a different etiology along with distinct immune responses [17,18,19,20]. Th1 and Th17 types of immune responses are frequently activated in psoriasis, whereas Th2 type immune response is activated in atopic dermatitis [17,18,19]. Atopic dermatitis often occurs in infants and follows a chronic relapse with severe immune responses and psoriasis pathogenesis commonly occurs in the aged population [21]. Topical steroids are one of the major dermatitis therapeutics. However, adverse effects of topical steroid-containing cream have been well established [22,23]. Although newer drugs have been developed for dermatitis, high cost is one of the major burdens while dermatitis patients are increasing every year [24,25]. According to recent reports, glucose metabolism is activated in the lesion of psoriasis and ablation of glucose transporter (Glut1) attenuates psoriasis-like symptoms in animal models [26,27]. Moreover, expression levels of high Km glucose transporter Glut2 are upregulated in the lumen of sweat glands of atopic dermatitis patients [28]. These reports indicated that glucose metabolism may be associated with dermatitis. Therefore, we determined whether glucose metabolism could be a target for dermatitis treatment.

## 2. Materials and Methods

### 2.1. Experimental Animals

For OXA-induced animal model of dermatitis, 7-week-old BALB/c mice were purchased from the Central Laboratory Animals (Seoul, Korea) and used after 1 week of quarantine. For 12-o-tetradecanoylphorbol-13-acetate (TPA)-induced animal model of dermatitis, 7-week-old C57/BL6 mice were purchased from Nara Biotech (Seoul, Korea) and used after 1 week of quarantine. Mice were housed in animal cages with controlled environmental conditions as temperature (20 ± 2 °C)/humidity (50% ± 5%) and maintained under specific pathogen-free conditions with 12 h light/dark cycle. All animals were cared for by using protocols approved by the Institutional Animal Care and Use Committee (Konkuk University, Republic of Korea). No. KU10160 (04 September 2019). All methods were performed in accordance with the relevant guidelines and regulations.

### 2.2. TPA-Induced Acute Dermatitis

Skin inflammation was induced in the mouse ear (*n* = 6) by topical application of 12-o-tetradecanoylphorbol-13-acetate (TPA). A total of 20 µL of TPA solution (50 µg/mL of TPA in 1%DMSO/99% acetone) was applied to the anterior and posterior surfaces of the mice ears every day for 4 days. Dexamethasone (0.4 mg/kg) and 2DG (50 mg/kg) were topically administrated to mice ears 1h after TPA treatment and the control group was the treated vehicle (1% DMSO/99% acetone). To determine inflammation, ear thicknesses were measured prior to each TPA application using a digital caliper (Mitutoyo, Tokyo, Japan) on days 0, 2, and 4. After four consecutive days, mice were sacrificed and 5mm-diameter ear biopsies were obtained with a punch (Kai Industries, Gifu, Japan). Ear biopsies were weighed and collected for histopathological analysis. All experimental procedures were approved by the Institutional Animal Care and Use Committee of the Konkuk University (KU19160).

### 2.3. OXA-Induced Animal Model of Dermatitis

Mice were divided into four groups (*n* = 3). Negative control was sensitized and challenged with phosphate-buffered saline (PBS). OXA group was treated with oxazolone (4-Ethoxymethylene-2-phenyl-2-oxazolin-5-one; Sigma-Aldrich, St. Louis, MO, USA). For therapeutic groups, 0.68 mg/kg of dexamethasone (DEX) and 150 mg/kg of 2DG were applied on the dorsal back 1 h after OXA challenge. The protocol of OXA-induced model has been described previously in detail, but with some modifications [29]. Briefly, mice were topically applied on the dorsal skin for sensitization with 100 μL of 1% OXA dissolved in acetone (Merck, Kenilworth, IL, USA) on day 0, and challenged with 0.2% OXA 3 days a week from day 7 to 14. All OXA apply and drug administration was performed under light anesthesia with isoflurane. Mice were photographed by digital single-lens reflex camera (F5.6 1/40, ISO800; Canon, Tokyo, Japan) on days 0, 7, and 14. Dorsal skins were used for staining of H&E and toluidine blue. All experimental procedures were approved by the Institutional Animal Care and Use Committee of the Konkuk University (KU19160).

### 2.4. AD Scoring

To visualize the severity of clinical dermatitis in AD model was scored for each item at 0, 7, and 14 days. Clinical symptom of AD including erythema/hemorrhage, scarring/dryness, edema, and excoriation/erosion were scored as follows: (0) none), (1) (mild, <20%), (2) (moderate, 20%–60%) and (3) (severe, >60%). The sum of the four individual scores was defined as the dermatitis severity score [30,31].

### 2.5. Histology

The ear and dorsal skin were collected using 5-mm biopsy punches (KAI Medical, Gifu, Japan) and fixed in 10% formaldehyde solution. Tissues were processed using standard methods (from 70% to 100% ethanol and xylene step) and were embedded in paraffin. Tissues were sectioned into 4µm and then stained by H&E and toluidine blue. The stained tissues were observed at 200X magnification under a light microscope (Olympus, CKX41, Tokyo, Japan). Pictures were taken using an image acquisition system (DP2- SAL; Olympus, Tokyo, Japan). Image analysis was calculated as the average of selected three random fields per each mouse. To observe morphology, H&E staining was performed. Tissue slides were de-paraffinized using xylene and hydrated using ethanol in decreasing concentrations (100%, 90%, 80%, and 70%), stained with Harris hematoxylin (Youngdong diagnostics, Youngin, Korea) and Eosin (Sigma-Aldrich, Kenilworth, IL, USA). Next, tissue slides were dehydrated by reverse step of ethanol and xylene, and mounted by Eukitt^®^ Quick-hardening mounting medium (Sigma, USA). Epidermal thickness was measured using ImageJ software program. Toluidine blue staining was used to count infiltrated mast cells into the dermis. Toluidine blue staining was done as previously reported with slight modification [32]. Briefly, hydrated tissue sections were stained with 0.1% Toluidine Blue O in 1% sodium chloride solution (pH 2; Sigma, USA) for 1min. Consequently, sections were washed with deionized water and briefly washed each three times with 95% and 100% ethanol for dehydration, then sections were cleared in xylene three times, and then sealed using Eukitt^®^ Quick-hardening mounting medium (Sigma, USA).

### 2.6. Cells and Reagents

NIH3T3/NFκB-luc cell line was purchased from Panomics (RC0015). HaCaT Cells were transfected with lentivirus generated from 293T cell by transfecting plasmids including pEZX-LvPG04 (HPRM36883-LvPG04, GeneCoporia), VSVG and delta8.2. Infected cells were selected puromycin. NIH3T3/NFκB-luc cell line maintained with FBM media (CC-3132, Lonza). HaCaT-luc cell line was maintained EpiLife^®^ media with HKGS (Human Keratinocyte Growth Supplement). Cells was maintained in a humidified incubator at 37 °C and 5% CO_2_. Recombinant Human TNF-alpha was purchased from Peprotech (300-01A-10). Bay was purchased from Sigma (11-7082).

### 2.7. Cell Viability Assay

Viability test was performed as previously described with slight modification [33]. Briefly, 1 × 10^4^ cells were plated in a 96-well plate. Then, 8h after 2DG treatment, cells were incubated with mixture (1:10) of EZ-Cytox cell viability assay kit (Dogen, EZ3000) and Fibroblast Growth Basal Medium (CC-3131, Lonza). Then plate was incubated for 30min in the incubator and determined absorbance at 450 nm with reference to 655 nm wavelength (iMark, Biorad).

### 2.8. Luciferase Assay

Luciferase assay was performed as previously described with slight modification [34]. First, 1 × 10^4^ cells were seeded in 96-well plates treated 25 ng/mL TNF-α for 8 h with or without 2DG. Cells were harvested and cell extracts were prepared using 60 μL of passive lysis buffer (Promega). Luciferase activities were measured using Veritas Luminometer (Turnur Designs, Sunnyvale, CA, USA).

### 2.9. Web-Based Meta-Analysis

Microarray datasets from studies (GSE120721 [35], GSE60709 [36], GSE121212 [37], GSE6012 [38], GSE107361 [39], GSE36842 [40], GSE5667 [41], and GSE46239 were analyzed using GEO2R (https://www.ncbi.nlm.nih.gov/geo/geo2r) to determine the levels of glucose transporters as well as glycolytic enzymes. Microarray datasets are a series of comparison results between skin biopsies from atopic dermatitis patients with control subjects.

### 2.10. Statistical Analysis

All statistical evaluations were performed using Prism 6 (GraphPad Software, La Jolla, CA, USA). Data are given as mean ± standard error of the mean (SEM). Statistical significance was analyzed using Student’s *t*-test and one-way ANOVA. *p* values of <0.05, <0.01, and <0.001 were considered as statistically significant differences.

## 3. Results

### 3.1. The Levels of Enzymes Associated with Glucose Signaling are increased in Dermatitis Patients

**Figure 1:**
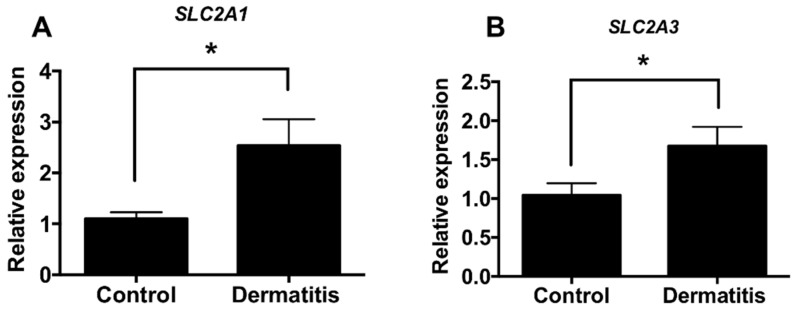
The expression levels of glucose transporters are increased from dermatitis patients. (**A**) Relative expression levels of Glut1 (*SLC2a1*) in normal controls (*n* = 38) and dermatitis patients (*n* = 34). (**B**) Relative expression levels of Glut3 (*SLC2a3*) in normal controls (*n* = 82) and dermatitis patients (*n* = 83) Data shown are mean ± SEM and analyzed by Student’s *t*-test (* *p* < 0.05).

We determined the expression levels of glucose transporters Glut1 (*SLC2a1*) and Glut3 (*SLC2a3*) in skin biopsies of dermatitis patients and control subjects from several datasets. Interestingly, we found that the expression levels of glucose transporters are increased in the lesion of atopy patients compared with control subjects (GSE120721 [35], GSE107361 [39], GSE36842 [40], GSE46239, GSE5667 [41], and GSE121212 [37]) (Figure 1A,B). Moreover, we found the levels of enzymes associated with glucose metabolism are highly increased in lesion of atopy patients compared with control subjects (GSE120721 [35], GSE107361 [39], GSE36842 [40], GSE46239, GSE5667 [41], GSE121212 [37]) (Tables S1 and S2). These results suggested that the expression levels of enzymes associated with glucose metabolism are increased in the lesion of dermatitis patients.

### 3.2. 2DG Ameliorates Acute Dermatitis Phenotype in TPA-Induced Animal Model

**Figure 2:**
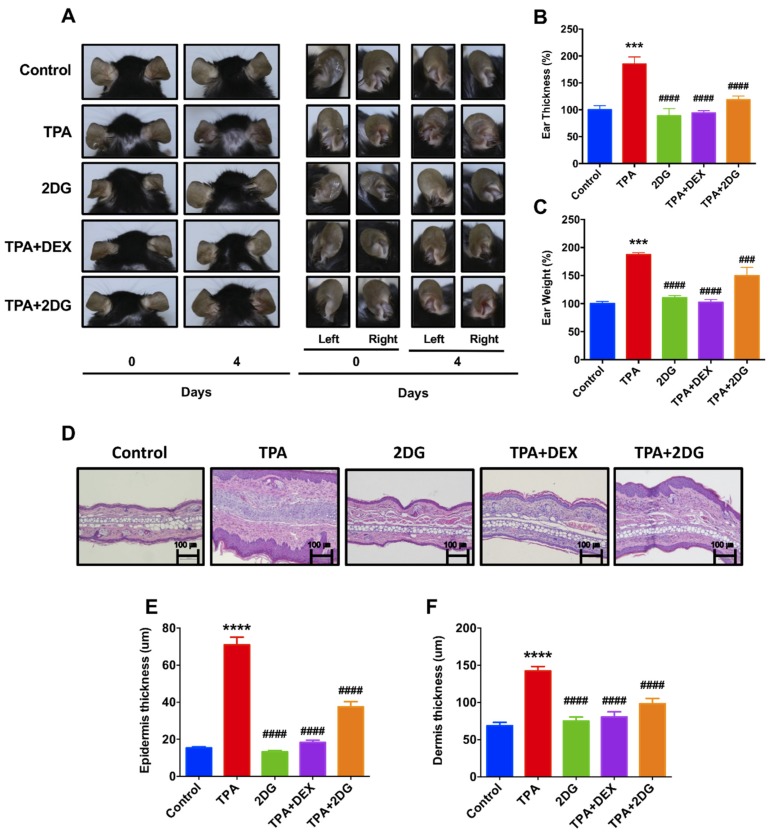
2-deoxy-d-glucose (2DG) alleviates 12-o-tetradecanoylphorbol-13-acetate (TPA)-induced animal model of dermatitis. (**A**) Representative images of mouse ears of anterior and posterior surfaces at 0, 2, and 4 days. Control: Control group, TPA: TPA-treated group, 2DG: 2-deoxy-d-glucose treated group, TPA + DEX: TPA and Dexamethasone treated group, TPA + 2DG: TPA and 2-deoxy-d-glucose treated group. (**B**) The levels of ear swelling were determined after mice sacrifice. (**C**) The levels of ear weight were determined after mice sacrifice. (**D**) H&E-stained sections of mouse ears. Original magnification = ×200. Scale bar = 100 μm. (**E**) Mean of epidermal thickness was measured using three different sections of each mouse (*n* = 3). (**F**) Mean of dermal thickness was measured using three different sections of each mouse (*n* = 3). Data are presented as mean ± SEM of changes in values. *** *p* < 0.01, **** *p* < 0.005 compared to control, ### *p* < 0.01, #### *p* < 0.005 compared to TPA.

In order to investigate whether inhibition of glucose metabolism attenuates dermatitis, we used 2DG, a glucose analogue, as a glycolytic inhibitor [42]. To determine whether 2DG attenuates dermatitis in animal model, we employed a 12-o-tetradecanoylphorbol-13-acetate (TPA)-induced mice model. Interestingly, mice cotreated with TPA with 2DG have shown less redness on ears compared to mice treated with TPA alone (Figure 2A). The levels of ear thickness and weight were robustly decreased in mice treated of TPA with 2DG compared to TPA-treated mice (Figure 2B,C). We found huge edema sites in TPA-treated mice ears and TPA-induced edema was attenuated by 2DG (Figure 2D). We also found that epidermis and dermis thickness are decreased in mice cotreated of TPA with 2DG by histological analysis (Figure 2E,F). These results indicated that topical administration of 2DG ameliorates TPA-induced inflammation.

### 3.3. 2DG Ameliorates Oxazolone-Treated Animal Model of Dermatitis

**Figure 3:**
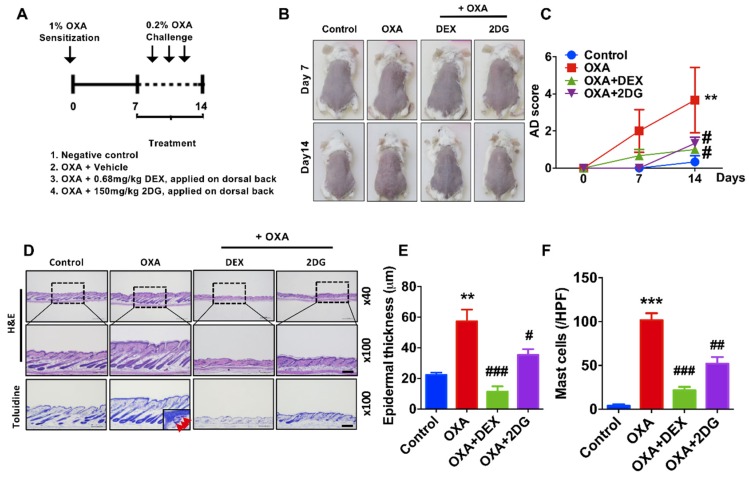
2DG attenuates dermatitis in oxazolone (OXA)-induced animal model. (**A**) Schematic diagram of OXA-induced animal model. Four groups: untreated controls, OXA only and mice treated with DEX (Dexamethasone) or 2-dexoy-d-glucose (2DG) 1 h after every OXA challenge (*n* = 3 per group). (**B**) Representative photographs of mouse ears from each group on day 14. (**C**) AD score was measured during treatment. (**D**) H&E staining and toluidine blue staining in dorsal skin lesions (**E**) Epidermal thickness (μm) was determined by micrometer. (**F**) Mast cells (red arrow) in dermis were counted. Scale bar, 200μm. Data are presented as mean ± SEM of changes in values. ** *p* < 0.01, *** *p* < 0.005 compared to control, # *p* < 0.05, ## *p* < 0.01 and ### *p* < 0.005 compared to OXA.

As we found that 2DG is effective on TPA-induced dermatitis, we further determined whether 2DG alleviates atopic dermatitis in animal models. Oxazolone (OXA) is widely used in animal model atopic dermatitis [43]. Redness of mice back treated with oxazolone was alleviated by coadministration of 2DG (Figure 3B). AD score was significantly decreased in mice cotreated with OXA with 2DG (Figure 3C). Furthermore, we found the levels of epidermis thickness are decreased in mice cotreated of OXA with 2DG by histological analysis (Figure 3D,E). Infiltrated mast cells into dermis were decreased in mice cotreated of OXA with 2DG (Figure 3F). These results indicated that administration of 2DG ameliorates oxazolone-induced dermatitis in mice models.

### 3.4. NFκB Activity is Not Regulated by 2DG in Keratinocytes and Fibroblasts

**Figure 4:**
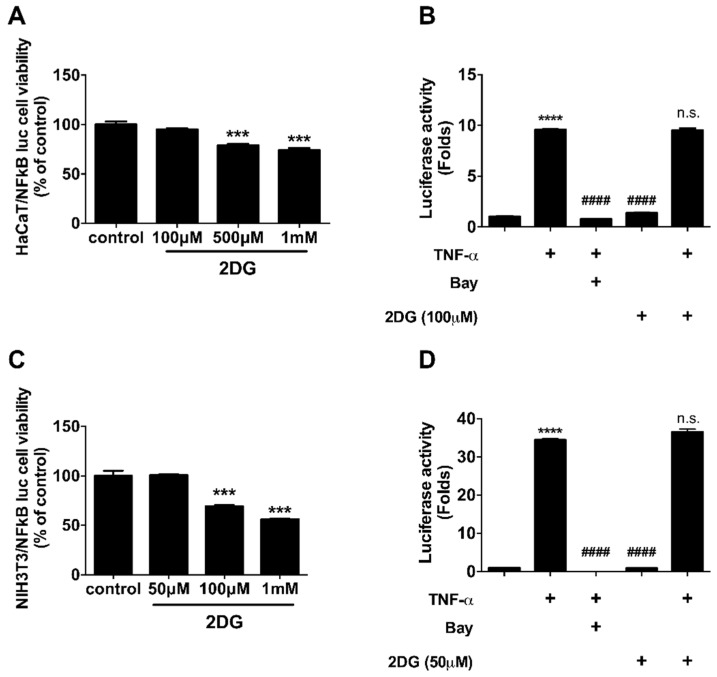
2DG is not able to modulate NFκB activity in skin cells. (**A**) Cellular viability of HaCaT cells was measured after 8 h of indicated 2DG treatment. (**B**) Luciferase activity was measured in HaCaT cells after 8 h of 100 μM of 2DG in the presence of TNF-α. (**C**) Cellular viability of NIH3T3 cells was measured after 8 h of indicated 2DG treatment. (**D**) Luciferase activity was measured in HaCaT cells after 8 h of 50 μM of 2DG in the presence of TNF-α. Data are presented as mean ± SEM of changes in values. *** *p* < 0.005 compared to control, **** *p* < 0.001, #### *p* < 0.005 compared to TNF-α and n.s., non-significant compared to TNF-α.

To determine the molecular mechanism of 2DG-mediated anti-inflammation in dermatitis, we investigated whether 2DG attenuates NFκB activation, which serves an important role in inflammatory signaling. We used 3T3 murine fibroblasts and HaCaT keratinocytes, which stably expressed luciferase reporter plasmid encoded with NFκB-binding motif. Prior to luciferase assay, we determined optimal concentration of 2DG using viability assay. In HaCaT and 3T3 cells 100 and 50 μM of 2DG were used for luciferase assay (Figure 4A,C). We found 2DG is not able to modulate NFκB activity in HaCaT and 3T3 cells (Figure 4B,D). These results indicated that NFκB might not be the primary molecular signaling pathway for 2DG-mediated anti-inflammatory signal in skin cells.

## 4. Discussion

We found the expression levels of glycolytic enzymes are increased in the lesion of dermatitis patients (Figure 1, Tables S1 and S2). Although further studies are required to determine whether glucose metabolism is activated in the lesion of dermatitis patients, we assume there is an abnormal metabolic shift in dermatitis patients according to recent publications [26,28,44]. Especially, glucose metabolism is activated in animal model of psoriasis and ablation of glucose transporter (Glut1) alleviates dermatitis symptoms in animal models of psoriasis [26]. This report supports that there is, at least in certain skin diseases, an abnormal metabolic shift including anaerobic glycolysis. We used 2DG, a glucose analogue that inhibits glucose metabolism whether 2DG alleviates TPA-induced inflammation in mouse ears (Figure 2). TPA-induced skin inflammation is usually used as a model for irritant contact dermatitis or psoriasis along with activation of Th1 and Th17 immune responses [45,46]. We found that the levels of mice ear thickness and epidermis are decreased by topical administration of 2DG (Figure 2B). Epidermis thickness is one of primary indicators of viability of keratinocytes and increased levels of epidermis thickness are frequently found along with high rate of keratinocyte proliferation [47]. We found that 500 μM 2DG inhibits keratinocyte viability in vitro (Figure 4A). Thus, 2DG is able to inhibit keratinocyte proliferation directly. Whereas, there was no toxicity found in keratinocytes from 2DG treated mice using histological analysis (Figure 2D). According to a recent report, mast cell-derived histamine induces keratinocyte proliferation in AD [48]. As 2DG inhibits mast cell infiltration (Figure 3F), 2DG may negatively regulate keratinocyte proliferation indirectly in animal models of dermatitis. Glucose signaling is important for redox metabolism and cytokine induction in keratinocytes [49]. As NFκB signaling is one of the primary modulators in inflammation, we determined whether 2DG attenuates NFκB activation in skin cells including keratinocytes and fibroblasts. We found 2DG treatment is not able to modulate NFκB activity in skin cells (Figure 4B,D). These data indicated that skin cells might not be primarily cells that are responsible for 2DG-mediated anti-inflammation in atopic dermatitis. On the other hand, inhibition of glucose and glutamine metabolism has been extensively studied in modulating immune response [8]. Moreover, 2DG suppresses immune cell activation and reduces disease severity in an autoimmune model of rheumatoid arthritis [50]. These observations suggested that 2DG primarily modulates immune response although rigorous study remains to elucidate the molecular mechanism. Oxazolone-induced dermatitis is an atopic dermatitis animal model, along with robust Th2 immune response [51]. We measured mast cells infiltration, which is one of the indicators of Th2 immune response. Moreover, histamine, released by mast cells serves a crucial role in promoting atopic march through vicious itch-scratch cycle [52]. We speculated that Th2 immune response is attenuated by 2DG treatment according to inhibition of infiltrated mast cells. As 2DG is widely investigated as chemotherapy for cancer treatment, we tested the adverse effect of 2DG in an animal model. We used 50 mg/kg of 2DG in a TPA-induced mice model, which is 10-fold lower compared to the study that shows an anticancer effect of 2DG [53]. The 50 mg/kg of 2DG treated mice had no adverse effect and were similar to normal mice (Figure 2). The confidence limit of 2DG concentration is 5100–126,000 mg/kg [9]. Previously, 1–5% 2DG was used topically for inhibition of herpes simplex virus and there was no report on keratinocyte apoptosis as well as mortality in mice and guinea pigs [10]. Moreover, 45 mg/kg of 2DG is well tolerated at the phase II clinical study [54]. These results suggest that 2DG could be one of the primary candidates as a repurposing drug although future studies are required to determine the appropriate dose of treatment for dermatitis therapeutics. In this manuscript, we found that the levels of enzymes associated with glucose metabolism are increased from skin biopsies of atopic dermatitis patients. We demonstrated that 2-deoxy-d-glucose (2DG), a glycolysis inhibitor, administration attenuates dermatitis in mice models. Overall, this is the first study showing that 2DG alleviates an animal model of dermatitis and further studies are required to investigate the molecular mechanism of the 2DG-mediated anti-inflammatory effect on dermatitis.

## Data Availability

All study data are available from the corresponding author upon request.

## Supplementary Materials

Supplementary materials can be found at https://www.mdpi.com/2227-9059/8/2/20/s1.

## Abbreviations

Glut	Glucose transporter
2DG	2-deoxy-d-glucose
DEX	Dexamethasone
OXA	Oxazolone
TPA	12-o-tetradecanoylphorbol-13-acetate
